# Quantitative Proteomic and Network Analysis of Differentially Expressed Proteins in PBMC of Friedreich’s Ataxia (FRDA) Patients

**DOI:** 10.3389/fnins.2019.01054

**Published:** 2019-10-14

**Authors:** Deepti Pathak, Achal Kumar Srivastava, M. V. Padma, Sheffali Gulati, Moganty R. Rajeswari

**Affiliations:** ^1^Department of Biochemistry, All India Institute of Medical Sciences, New Delhi, New Delhi, India; ^2^Department of Neurology, All India Institute of Medical Sciences, New Delhi, New Delhi, India; ^3^Department of Paediatrics, All India Institute of Medical Sciences, New Delhi, New Delhi, India

**Keywords:** Friedreich’s ataxia, PBMCs, proteomics, 2D-DIGE, PDHE1, ACTC1, protein–protein interaction

## Abstract

Friedreich’s ataxia (FRDA) is an autosomal recessive neurodegenerative disorder caused by an expanded (GAA) trinucleotide repeat in the FXN gene. The extended repeats expansion results in reduced transcription and, thereby, decreased expression of the mitochondrial protein, frataxin. Given the ongoing drug trials, identification of reliable and easily accessible biomarkers for monitoring disease progression and therapeutic intervention is a foremost requirement. In this study, comparative proteomic profiling of PBMC proteins from FRDA patients and age- and gender-matched healthy controls was done using 2D-Differential in-Gel Electrophoresis (2D-DIGE). Protein–protein interaction (PPI) was analyzed using BioGRID and STRING pathway analysis tools. Using biological variance analysis (BVA) and LC/MS, we found eight differentially expressed proteins with fold change ≥1.5; *p* ≤ 0.05. Based on their cellular function, the identified proteins showed a strong pathological role in neuroinflammation, cardiomyopathy, compromised glucose metabolism, and iron transport, which are the major clinical manifestations of FRDA. Protein–protein network analysis of differentially expressed proteins with frataxin further supports their involvement in the pathophysiology of FRDA. Considering their crucial role in the cardiac and neurological complications, respectively, the two down-regulated proteins, actin α cardiac muscle 1 (ACTC1) and pyruvate dehydrogenase E1 component subunit β (PDHE1), are suggested as potential prognostic markers for FRDA.

## Introduction

Friedreich’s ataxia (FRDA) is an early-onset neurodegenerative disorder primarily caused by homozygous (GAA) repeat expansion in the first intron of the frataxin gene (*FXN*), located on chromosome 9q21.1 (Campuzano et al., 1996). FRDA is characterized by progressive degeneration of large sensory neurons in the dorsal root ganglia (DRG) and spinocerebellar tract and cerebellar atrophy (dentate nucleus) (Pandolfo and Pastore, 2009; Koeppen et al., 2013). Clinically, FRDA is manifested through gait and limb ataxia, deep sensory loss, dysarthria, muscular weakness, positive extensor plantar response, scoliosis, and pes cavus (Harding, 1981; Pandolfo and Pastore, 2009). Non-neurological symptoms, principally hypertrophic cardiomyopathy and diabetes mellitus, often are observed in later stages of the disease (Harding and Hewer, 1983; Peverill et al., 2018). By and large, the number of GAA repeats in FRDA patients ranges from 44 to 1700 repeats, with repeat numbers between 600 and 900 being the most common (Cossée et al., 1997). However, in less than 5% of the cases, patients show compound heterozygous nature, with GAA expansion in one allele and “loss of function” point mutation in the other (Cossée et al., 1999).

The allele containing long (GAA) repeats is very vulnerable to adopt unusual non-B DNA structures such as intra-molecular triplexes and sticky DNA (Rajeswari, 2014; Wells et al., no date) or DNA–RNA hybrid structures like R-loops (Grabczyk et al., 2007; Bidichandani and Delatycki, 2018). It is also known that expanded GAA repeats are liable to heterochromatinization by hypoacetylation of histones H3 and H4 and hypermethylation of lysine 9 in histone H3 (Saveliev et al., 2003). Such abnormal DNA structures obstruct the RNA polymerase II, repressing the transcription of *FXN* gene, which encodes an essential mitochondrial protein, frataxin. Transcriptional repression results in reduction of frataxin levels by 30–70% in FRDA patients (Campuzano et al., 1997; Gottesfeld et al., 2013). It is also well established that the higher the (GAA) repeat number, the lower is the frataxin level and earlier is the onset of the disease (Campuzano et al., 1996; Pandolfo and Pastore, 2009; Gerhardt et al., 2016).

Frataxin is a highly conserved protein found in prokaryotes and eukaryotes. It is synthesized as a 210-amino-acid precursor and proteolytically processed in mitochondria to form 14 kDa mature form (Koutnikova et al., 1998; Cavadini, 2002; Pandolfo and Pastore, 2009). Frataxin is ubiquitously, but differentially, expressed in all mitochondria containing cell types, with expression being highest in metabolically demanding tissues of the spinal cord and heart, and intermediate in liver, skeletal muscle, cerebellum, and pancreas (Koutnikova et al., 1997; Bencze et al., 2006). Although the exact function of frataxin remains unclear, however, it is now understood that frataxin plays a major role in mitochondrial iron metabolism including iron binding, iron storage, iron–sulfur cluster (ISC) biosynthesis, and potential defense against reactive oxygen species (ROS) (Adinolfi et al., 2002; Bencze et al., 2006; Gakh et al., 2006; Richardson et al., 2010). Thus, reduced levels of frataxin results in decreased ISC biosynthesis, increased sensitivity to oxidative stress, and increased mitochondrial iron (Foury and Cazzalini, 1997; Rötig et al., 1997). In our recent study, we have reported that the mitochondrial iron accumulation in FRDA occurs at the expense of circulating iron, resulting in decreased levels of plasma iron in patients as compared to healthy individuals (Pathak et al., 2019).

Given the past studies and ongoing research, our understanding of the pathogenesis of FRDA and its therapeutics has fairly widened. However, the need of a reliable biomarker that can measure the disease progression and monitor drug effects is still lacking. Clinical parameters are being used as biomarker in drug trials, but their patient-to-patient variability and poor ability to detect slow progression of this disorder make them unsuitable as biomarkers in the long run (Bürk et al., 2009).

The Food and Drug Administration defines biomarker as “objectively measurable characteristic that is indicator of physiological and pathological processes or reflects response to therapeutic interventions” (Biomarkers Definitions Working Group, 2001). Frataxin, being the center of the disease pathogenesis, is therefore an obvious and strong candidate for FRDA diagnosis (Bencze et al., 2006). However, the level of frataxin is highly variable even in healthy individuals, making it difficult to determine a baseline value (Boehm et al., 2011). Furthermore, the recent clinical trials of frataxin up-regulating drugs have shown to increase the frataxin levels but with no consequent improvement of neurological and other clinical symptoms (Sturm et al., 2010; Boesch et al., 2014; Marcotulli et al., 2016). It is therefore, highly debatable and controversial whether frataxin can be a suitable biomarker for FRDA. Thus, the identification of reliable and easily accessible biomarkers for monitoring disease progression and therapeutic intervention becomes a foremost requirement.

Because of its extensive protein pool, peripheral blood mononuclear cells (PBMCs) have emerged as potential surrogate tissue to search for biomarkers using proteomic techniques. PBMCs can monitor subtle physiological changes that are difficult to detect in plasma samples, especially those related to immune system. Lack of highly abundant masking proteins further adds to its advantage over plasma proteome. Therefore, this present study was aimed at PBMC proteomics in search of alternate protein biomarkers for FRDA.

## Materials and Methods

### Patients Recruitment

Clinically suspected FRDA patients were recruited from the Department of Neurology and Department of Paediatrics, All India Institute of Medical Sciences (AIIMS), New Delhi, India. Homozygous FRDA patients carrying expanded (GAA) repeats in both alleles were recruited in this study (*n* = 25). Age- and gender-matched individuals with no past history of any neuromuscular symptoms and with no familial relation with patients were enrolled as controls (*n* = 25). Patients showing overlapping symptoms of more than one kind of neurological disorder, or under any therapeutic interventions, were excluded from the study. Clinical severity of disease was assessed using Friedreich Ataxia Rating Scale (FARS) scoring. Written consent was taken from each subject at the time of their enrolment in this study.

### Ethics Statement

Ethical clearance (IESC/T-45/21.01.2015) was obtained as per institutional ethical committee guidelines from AIIMS, New Delhi.

### Isolation of PBMCs

Peripheral blood sample (10 ml) from each patient and healthy control was drawn into K_2_ EDTA vacutainer tubes (BD Biosciences, San Jose, CA, United States). PBMCs were isolated using Ficoll density gradient centrifugation method. Blood was diluted 1:1 with 1 × phosphate buffered saline (PBS) prior to transferring it into the ficoll tubes. After centrifugation (20 min, 1000 × *g*, and room temperature), the buffy coat of PBMC cells was pooled and transferred into a 15-ml falcon. PBMCs were then washed twice by diluting with 10-ml PBS and centrifuged again for 10 min at 250 × *g*. The obtained cell pellets were stored at −80°C until further protein and genomic DNA extraction (Qiamp blood DNA mini kit, Qiagen GmbH, Germany).

### Genetic Analysis for (GAA) Repeats

Long-range PCR Kit containing LR-PCR buffer, dNTPs, and long-range Taq polymerase and a set of primers were obtained from Thermo Fisher Scientific, United States. The PCR amplification for all the samples was done twice on an MJ Mini Thermocycler (Bio-Rad Laboratories, United States). Forward primer, 5′GGAGGGATCCGTCTGGGCAAAGG-3′ and reverse primer 5′CAATCCAGGACAGTCAGGGCTTT-3′ were used in PCR master mix with the following cycling conditions: 94°C for 20 s, 68°C for 2.5 min, followed by 17 cycles in which the length of the 68°C step was increased by 15 s/cycle (Swarup and Rajeswari, 2007; Swarup et al., 2013; Dantham et al., 2016, 2018). (GAA) repeat numbers were calculated by measuring the band size of amplified fragments on 1.2% agarose gel.

### Clinical Assessment by Friedreich Ataxia Rating Scale (FARS)

All genetically confirmed FRDA patients were assessed on FARS. FARS consists of quantified variants of the neurological exam based on gait functions, upper and lower limb activity, upright stability, and neurological examination such as bulbar and peripheral nerve functioning. It is broadly divided into three sub-scales, namely, ataxia score (6 points), activities of daily living ADL (36 points), and neurological examination (117 points), making a total of 159 points. FARS scale was developed by Subramony and colleagues to analyze the neurological changes to identify predictors of progression and generate power calculations for clinical trials (Lynch et al., 2006). Since then, it is the most used rating scale in clinical assessment of FRDA.

### Total Protein Extraction From PBMCs

PBMC pellets were resuspended in 500 μl of lysis buffer [7 M urea, 2 M thiourea, 4% CHAPS (3-cholamidopropyl dimethylammonio 1-propanesulfonate), 40 mM Tris-base, 1% dithiothreitol (DTT)] with 1% protease inhibitor and incubated on ice for 20 min followed by sonication. Samples were then centrifuged (25 min, 12,000 × *g*) and supernatant was discarded. The samples were then desalted by acetone precipitation followed by protein quantification using Bradford method.

### Estimation of Frataxin Levels Using ELISA

The comparative levels of frataxin proteins in PBMC cells of FRDA patients and healthy individuals were measured using human frataxin *in vitro* SimpleStep ELISA (Enzyme-Linked Immunosorbent Assay) kit (Abcam, United Kingdom). Briefly 25 μg of protein was added to immune affinity anti-tag antibody-coated 96-well plate with affinity tag labeled capture antibody and a reporter conjugated detector antibody that immune captured the sample analyte in solution. After a 1-h immunocapture incubation period, a signal was generated using a TMB–HRP reaction (3, 30, 5, 50-tetramethylbenzidine: horseradish peroxidase) solution that produced a colorimetric signal proportional to the amount of bound analyte (frataxin); the signal was measured at 600 nm.

### Cy Dye Labeling of PBMC Proteins With Cy Dyes

PBMC protein samples were labeled with fluorescent cyanine (Cy) dyes (GE Healthcare, Singapore). Six FRDA patients and age- and gender-matched healthy controls were selected for proteomics analysis (six biological replicates). The internal standard was prepared by combining equal amounts of each of the 12 samples. Prior to fluorescent dye labeling, the pH of each sample was adjusted to around 8. For each sample, 50 μg of proteins was labeled with 200 pmol of either Cy3 or Cy5, using minimal labeling method (GE Healthcare). Dye swapping was done to ensure no biasing of Cy dyes for any protein sample. Internal standard was labeled with 200 pmol of the Cy2 fluorophore in each experimental set (Table 1). The labeling was performed in the dark and on ice for 30 min. The reaction was stopped by adding 10 mM lysine and the samples were stored on ice for 15 min. Afterward, the Cy2, Cy3, and Cy5 sample were pooled in a single tube.

**TABLE 1:** Details of the experimental design for the DIGE experiments carried out for the comparative PBMC proteome between FRDA patients and control group.

**Experiment sets**	**Control group (50 μg)**	**FRDA (50 μg)**	**Pooled sample (1/6 of each sample from each set)(50 μg)**
Set1	*Cy5*	*Cy3*	*Cy2*
Set2	*Cy5*	*Cy3*	*Cy2*
Set3	*Cy5*	*Cy3*	*Cy2*
**Dye swapping**			
Set4	*Cy3*	*Cy5*	*Cy2*
Set5	*Cy3*	*Cy5*	*Cy2*
Set6	*Cy3*	*Cy5*	*Cy2*

### 2D Differential in Gel Electrophoresis (2D-DIGE)

The labeled proteins were separated in first dimension using immobilized pH gradient (IPG) strips (pH 4–7, 13 cm) (GE Healthcare), which were rehydrated in the dark for 14 h in rehydration buffer (7 M urea, 2 M thiourea, 20 mM Tris, 2% w/v CHAPS, 50 mM dithiothreitol, and 1% v/v IPG buffer, pH 4–7) containing pooled protein samples. Isoelectric focusing (IEF) was performed on an IPGphor 3 unit (GE Healthcare) until 32,000 VhT were reached, allowing maximal 50-μA current (at 20°C). The settings of the first dimension were as follows: 3 h at 50 V in step-n-hold, 1 h at 200 V in step-n-hold, 1 h at 1000 V in gradient, 1 h at 1000 V in step-n-hold, 1 h at 3000 V in gradient, 1 h at 3000 V in step-n-hold, 1 h at 6000 V in gradient, 3 h at 6000 V in step-n-hold. Before starting the second dimension, the strips were equilibrated by incubating them in SDS equilibration buffer 1 (6 M urea, 30% glycerol, 2% SDS, and 50 mM Tris-HCl, pH 8.8) containing 1% dithiothreitol DTT for 15 min. This was followed by incubating the strips in SDS equilibration buffer with 4% iodoacetamide and bromophenol blue again for 15 min. The equilibrated strips were then embedded on top of 12.5% acrylamide gels and sealed with 1.0% (w/v) agarose followed by second-dimensional separation on an Ettan DALT-six electrophoresis system at 12 mA/gel settings (GE Healthcare).

### DeCyder Image Analysis

Typhoon 9400 scanner (GE Healthcare Life Sciences) was used to scan the gels after the completion of SDS run. The Cy2, Cy3, and Cy5 components of each gel were individually scanned at wavelengths of 488/520, 532/580, and 633/670 nm, respectively. After scanning, the differential analysis of the gels was performed in Decyder software 7.0 (GE Healthcare). The differential in-gel analysis (DIA) module was used for intra-gel spot detection, i.e., to compare the intensities of specific protein spots between FRDA samples with the internal standard within the same gel. The Cy2, Cy3, and Cy5 gel images were then merged and normalized spot volumes were calculated. The processed gels were then loaded into the biological variation analysis (BVA) module, which was then used to simultaneously match all 18 maps from the six DIGE gels from each FRDA patient, healthy control, and internal control by selecting one master gel. Comparative intensities of individual protein spots within the proteomes of control group and FRDA patients were collated into a single analysis using the BVA. Manual detection of the spot matching was done using land marking and re-matching as per the manufacturer’s protocol. Student’s *t*-test and one-way ANOVA were used to calculate significant differences in the relative abundance of individual protein spot features between FRDA patients and healthy controls. Protein abundance ratios larger than +1.5 or smaller than −1.5 were set as a threshold for significant changes.

### Trypsinization and Electrospray Ionization Mass Spectrometry (ESI-MS)

Coomassie brilliant blue (CBB)-stained portions of the gel containing protein spot of interest were excised, destained, and subjected to repeated dehydration rehydration steps prior to overnight digestion at 37°C with sequencing grade modified trypsin Gold (Promega, Madison, WI) as previously described (Kondo et al., 2006; Miyachi et al., 2017). After digestion, tryptic peptides were extracted with 45% acetonitrile/0.1% trifluoroacetic acid and concentrated with a vacuum evaporator (Speed-Vac; Thermo Electron, San Jose, CA). The resulting peptide mixture was separated by reverse-phase chromatography (Tempo TM nano-LC system; Applied Biosystems, Foster City, CA) using a Pep Map C18 column. The eluting peptides were ionized by electrospray ionization and analyzed by QSTAR XL system (Applied Biosystems) for peptide mass finger-printing (PMF). Nanospray ionization was carried out by using an ion spray voltage at 900 V. The progress of each run was monitored by recording the total ion current for positive ions as a function of time in the *m*/*z* range of 400–1600 for MS and 140–1600 for MS/MS. The spectra were acquired in an information-dependent manner utilizing the Analyst QS 2.0 software. The other parameters set were interface temperature, 50°C; curtain gas flow, 1.13 L/min; focusing potential, 280 V; declustering potential 2, 15 V.

### Protein Identification by PMF

Processed peak lists were submitted for PMF in Swiss-Protand NCBI database of *Homo sapiens* using Mascot version 2.6.2 (Matrix Science, Cambridge, United Kingdom). Oxidation of methionine and carbamidomethylation of cysteine were taken as fixed and variable modifications of peptides, respectively, for peptides with charged state from + 2 to + 3. The peptide mass tolerance range and fragment mass tolerance were both set to ± 0.3 Da. Those hits with (probability based) MOWSE score of more than the designated value (*p* < 0.05) were accepted and were successfully identified.

### Protein–Protein Network Analysis Construction

Biological General Repository for Interaction Datasets database (BioGRID, version 3.5.170 was used to identify the known protein interaction network of the protein of interest, frataxin^1^. Further, the complete PPI network of frataxin and eight differentially expressed proteins was constructed by the STRING network (Search Tool for the Retrieval of Interacting Genes/Proteins) (Version: 10.5^2^). To construct the PPI network in the STRING database, *H. sapiens* was selected as the organism and the option of multiple proteins was selected. Maximum number of first shell interaction was limited to the query proteins and minimum requirement interaction score was set to 0.150. Interaction combined score ≥0.4 was regarded as significant.

### RNA Isolation

Total RNA was isolated from PBMC by the TRIzol method (Invitrogen) in combination with RNeasy Mini Kit from Qiagen. The method combines phenol/guanidine based lysis and silicon membrane-based purification of total RNA. Briefly, 200 μl of PBMCs sample was homogenized in 1 ml TRIZOL (Invitrogen) using a bench-top homogenizer (Mixer Mill MM 301). Homogenate was separated into three phase by adding chloroform. The upper phase of clear liquid contained RNA and was used for RNA extraction. All the samples were run through an RNeasy Mini spin column where total RNA binds to the column membrane while phenol and other impurities are washed away. To remove contamination of DNA on-column, DNase digestion was done to confirm DNA free sample. RNA was eluted from the column using 30 μl of diethylpyrocarbonate (DEPC)-treated, RNase-free, water. Purity of RNA was confirmed spectrophotometrically and found to be suitable for further processing.

### cDNA Synthesis and Quantitative Real-Time PCR (qPCR) Analysis

The qPCR validation was done in 10 FRDA patients and 10 matched healthy controls in triplicate reactions. These samples were taken from different patients and control from those the proteomics results were obtained. Total RNA was reverse-transcribed into cDNA using a PrimeScript 1st strand cDNA Synthesis Kit (Takara Bio Inc.). The mRNA levels of four differentially expressed proteins, actin α cardiac muscle 1 (ACTC1), pyruvate dehydrogenase E1 subunit β (PDHE1), caspase 8 (CAS8), and serotransferrin (TF) were assayed. Primers designing for ACTC1, PDHA1, CAS8, and TF were performed using Gene Bank Graphics database^3^ and Primer3 software^4^. GAPDH was used as reference in order to normalize expression levels and to quantitate changes in gene expressions between the control and FRDA samples. The qPCR was run on the ABI Prism 7500 Sequence Detector (Applied BioSystems, Foster City, CA): 2 min at 50°C, 10 min at 95°C, and 40 cycles of 95°C for 15 s and 60°C for 1 min. Gene and primer set with details are given in Table 2. Changes in gene expression of the selected genes between FRDA and controls were determined by the comparative ΔΔCT method (Livak KJ, Schmittgen TD., 2001) using GAPDH as internal references.

**TABLE 2:** Forward and reverse primer sequences with amplicon length and melting temperature for genes validated by quantitative real-time PCR.

**Name of the gene**	**Forward (F) and reverse (R) primer sequences (5′-to-3′ direction)**	**Amplicon length (bp)**	**Melting temperature (°C)**
TF	F—GCTTACCTGGCTCCCAATAA R—CCACTATCCTTCTTCACCACAG	110	62 62
CAS8	F—TGCCTACAGGTTCCACTTCTG R—ATGATCGACCCTCCGCCAG	94	59.65 61.50
PDHE1	F—GTATGGATGAGGAGCTGGAAAG R—GCCCTCGACTAACCTTGTATG	93	62 62
ACTC1	F—CAGGGAGTTATGGTGGGTATG R—CCATGCTCGATGGGATACTT	101	62 62

## Results

**FIGURE 1:**
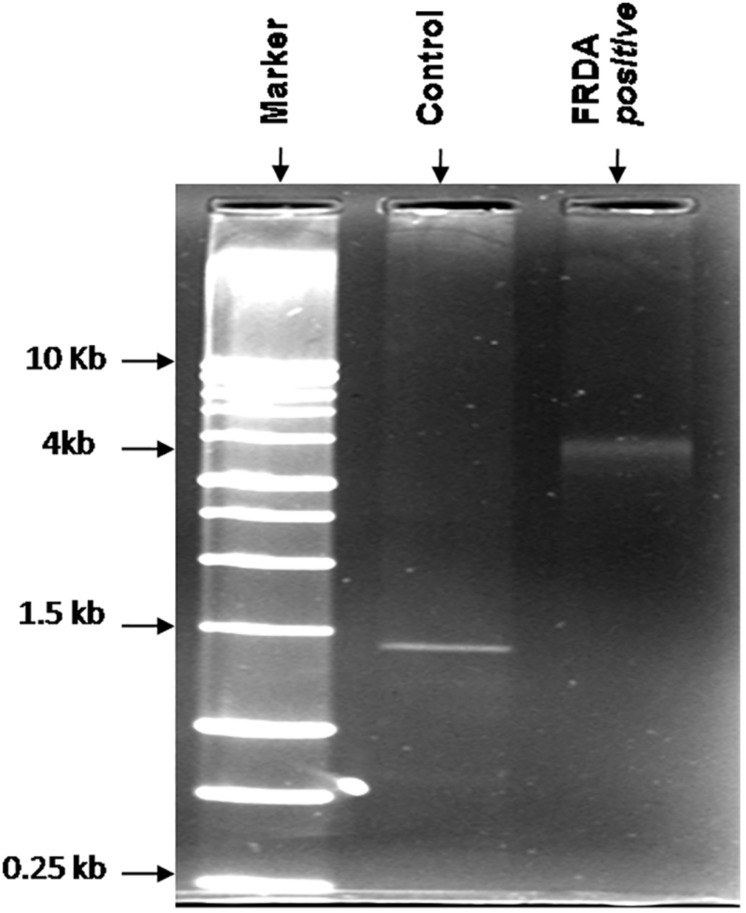
Representative image of the agarose gel of the PCR products obtained from FRDA patients and healthy controls. Lane 1, 1-kb DNA ladder; Lane 3, homozygous FRDA patient with 4-kb bands; and Lane 2, healthy control.

All suspected FRDA patients were genetically tested for (GAA) repeat expansions by long-range PCR method. Long PCR consists of auto extensions, i.e., the sequential increase in the time period of extension in all repeating cycle conditions. Genetic analysis of genomic DNA showed that 25 out of 86 suspected patients have pathogenic range of (GAA) alleles and was confirmed for FRDA. Genomic DNA of all healthy controls carry normal range 20 ± 4 (GAA) repeats (60 bp) producing a band size of ∼1.5 kb on 1.2% agarose gel. Size of (GAA) repeats in FRDA patients were found to be greater than 2 kb (above 200 repeats) as shown in Figure 1.

All FRDA-confirmed patients were consistent with the symptoms of gait and limb ataxia, dysarthria, and loss of lower limb reflexes. However, MRI reports suggested cerebellar atrophy in less than 50% of patients. Similarly, cardiomyopathy, scoliosis, and diabetes mellitus were also not regular symptoms in patients in this study. Further, the severity of disorder in patients was measured by clinical severity rating scale, FARS. Mean (± SD) FARS score of confirmed patients in this study patients was 68 ± 24.

**FIGURE 2:**
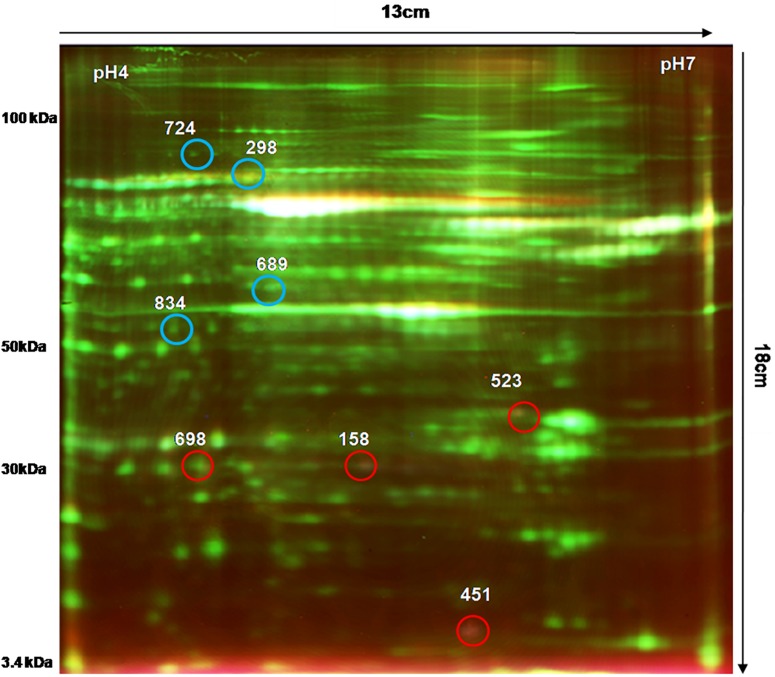
2D-DIGE image of PBMC proteins obtained from FRDA patients and healthy controls. Comparative PBMC proteomics of FRDA patients (*n* = 6) and age- and gender-matched healthy controls (*n* = 6) was done on 13-cm-long, pH 4–7 IPG strips using Cy3 (50 μg), Cy5 (50 μg), and pooled internal standard (50 μg). A total of 150 μg of total PBMC protein was used for isoelectric focusing (IEF) followed by second-dimension electrophoresis on SE 600 Ruby gel apparatus using 12.5% SDS-PAGE gel (16 × 13 cm) in dark. Gels were scanned by Typhoon variable mode imager for each Cy dye. Blue circles show protein spots that were up-regulated, and red circled spots were down-regulated in FRDA patients compared with matched healthy controls.

We analyzed the proteome variation in the PBMCs of FRDA patients as compared to age- and gender-matched healthy controls by 2D-DIGE. Each DIGE gel unfolds above 1000 protein spots in both control and patient gels (Figure 2). After BVA analysis of all 18 gels, results showed eight protein spots with fold change difference above 1.5-fold, and *p* < 0.05 and confidence > 90% (Table 3). Among the eight protein spots, four were found to be down-regulated and four of them were up-regulated. Mass spectrometry analysis using LC-MS/MS and homology search identified down-regulated proteins as PDHE1, alternate protein HNRNPUL2 (HNRNPUL2), myosin regulatory light chain 12A (MYL12A), and ACTC1 as down-regulated proteins. The identification of HNRNPUL2 gave a low score (43), and this perhaps because of the stringency in the searching parameters that are used and with a narrow window. Also the high peptide number (26) appears to be due to the presence of other proximate peptides taken erroneously during the excision of spot of interest. The up-regulated protein spots identified were TF, Sorbin and SH3 domain containing 1 isoform CRA (SORBS1), CASP8, and N-alpha-acetyltransferase 16 (NAA16). Table 4 shows the statistical results of Decyder analysis and Mascot search of all confirmed proteins. Further, ELISA assay showed a significantly decreased level of frataxin in the PBMC of FRDA patients (mean ± SD), 48 ± 26, as compared to control group (mean ± SD), 82 ± 36.

**TABLE 3:** List of differentially expressed PBMC proteins in FRDA patients analyzed by DeCyder software version 7.0.

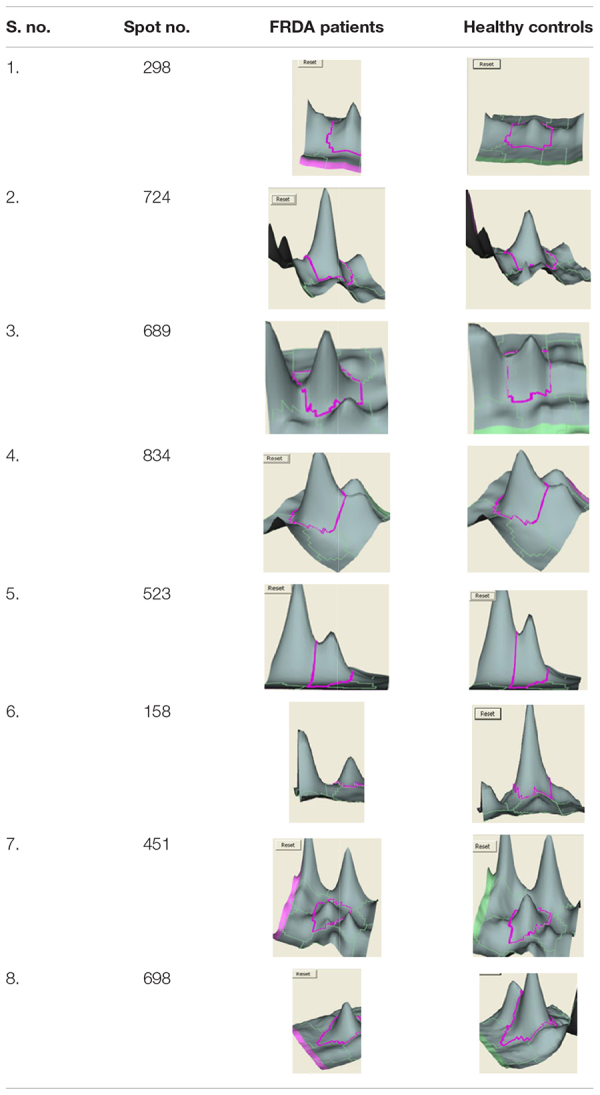

*Negative sign in column 2 represents down-regulation of that protein in FRDA patients as compared with matched healthy controls.*

**TABLE 4:** Differentially expressed PBMC proteins in Friedreich’s ataxia patients and their characterization using Mascot search and the proteins identified are given in the last column.

**Spot no.**	**Fold change**	***P*-value**	**pI**	**Mol wt (kDa)**	**MASCOT score**	**Accession no.**	**Coverage %**	**Matched peptide**	**Protein identified**
**Up-regulated proteins**									
298	1.90	0.048	5.8	90	78	TF_HUMAN	38	8	Serotransferrin precursor
724	4.17	0.028	4.2	92	63	SORB1_HUMAN	8	17	Sorbin and SH3 domain containing 1, isoform CRA
689	2.76	0.046	4.7	46	88	CASP-8_HUMAN	26	16	Caspase 8
834	2.61	0.049	4.6	58.9	53	NAA16_HUMAN	7	18	*N*-alpha-acetyltransferase 16
**Down-regulated proteins**									
523	–2.61	0.043	5.9	35.8	138	PDHE1B_HUMAN	32	14	Pyruvate dehydrogenase E1 component subunit β
158	–3.41	0.0026	4.1	70	43	HNRNPUL2_HUMAN	12	26	Alternate protein HNRNPUL2
451	–1.52	0.0056	5.8	20.3	81	MYL12A_HUMAN	9	12	Myosin regulatory light chain 12A
698	–4.92	0.038	4.3	31	101	ACTC1_HUMAN	22	12	Actin alpha cardiac muscle 1

**FIGURE 3:**
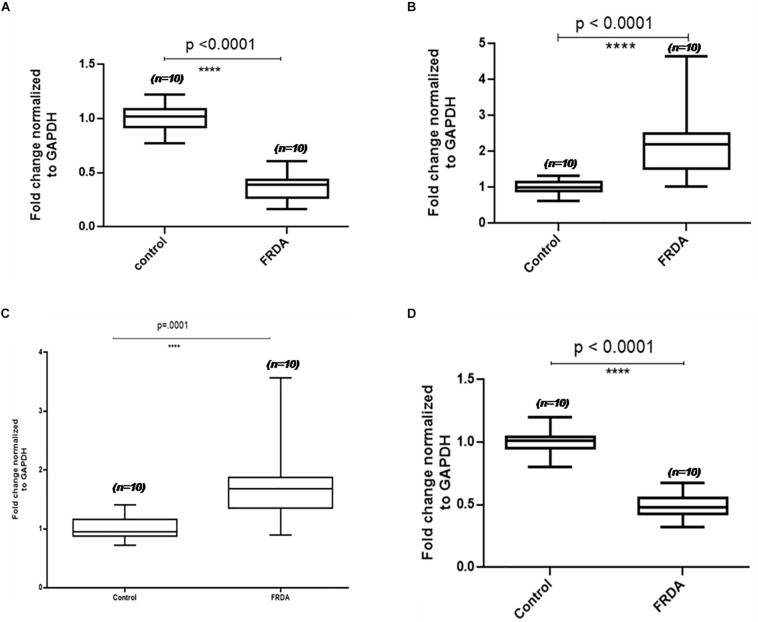
Validation results of four differentially expressed PBMC proteins in FRDA using qPCR. Box-and-whiskers plots of mRNA levels of **(A)** actin alpha cardiac muscle (ACTC1), **(B)** caspase 8 (Cas8), **(C)** serotransferrin (TF), and **(D)** pyruvate dehydrogenase subunit E1 (PDHE1) in PBMC of FRDA patients and controls. The ends of the whiskers show the position of the lowest and highest quartiles of the data while the edges and line in the center of the box show the upper and lower quartiles. Line inside the box represents the median value (*p* < 0.001) obtained using Mann–Whitney *U* test.

To validate the proteomics results, four proteins, two up-regulated and two down-regulated, were selected for qPCR analysis. The mRNA expression levels observed in qPCR were consistent with proteomics results. After GAPDH normalization, mRNA expression levels of PDHE1 were found to be approximately 2-fold decreased (2.6-fold down-regulated at the protein level) in patients as compared to the control group, and then RNA levels of ACTC1 were 3.3-fold decreased (4.9-fold down-regulated at protein level) in patients as compared to the control group, whereas the mRNA levels of TF and CASP8 were found to be 1.8- and 2.1-fold increased (1.9- and 2.7-fold up-regulated at the protein level) in patients as compared to the control group, respectively (Figure 3).

### Protein–Protein Interaction (PPI) Network Analysis

**FIGURE 4:**
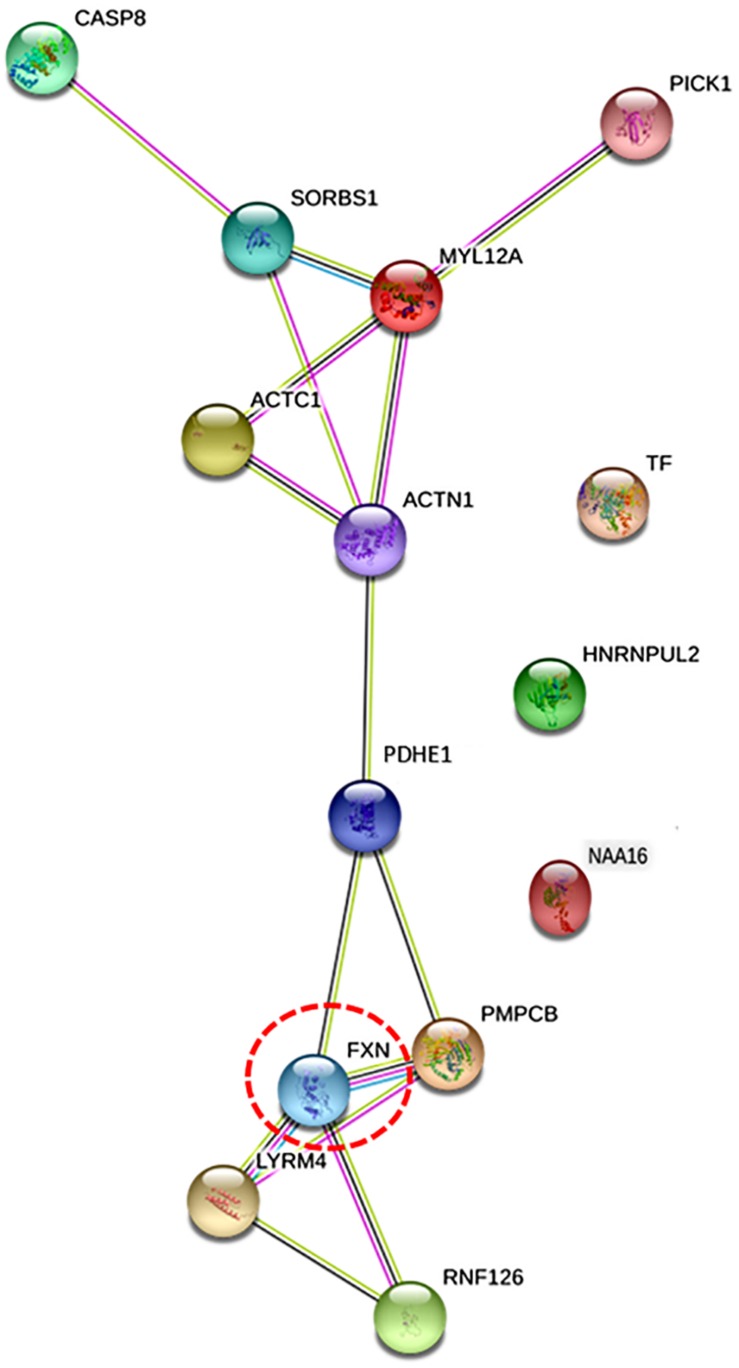
The figure illustrates protein network interaction of eight differentially expressed proteins identified with protein of interest, frataxin (*Fxn*) using STRING software. Proteins that were found to have direct interactions with frataxin are also included (LYRM4, PMPCB, RNF126, and ACTN1). Nodes denote genes/proteins. Frataxin is highlighted in red circle [protein interacting with C kinase (PICK1) was included in the network by software as a MYL12A interacting protein].

BioGRID database identified four frataxin interacting proteins, LYR motif containing 4 (LYRM4), peptidase mitochondrial processing β subunit (PMPCB), ring finger protein 126 (RNF126), and actin alpha 1 (ACTN1). Further, to investigate the intracellular communication of eight differentially regulated proteins (identified in proteomics study) with frataxin and four frataxin interacting protein (obtained from BioGRID), STRING PPI analysis was done. A network map was obtained, which depicted proteins of the highest interaction with FXN as the closer nodes while the subordinate proteins were arranged as distant nodes from FXN (Figure 4). The directly interacting partners for FXN are PMPCB, LYRM4, RNF126, and PDHE1, while indirectly interacting partners are ACTN1, ACTC1, MYL12A, SRBS1, and CASP8. PPI network showed that proteins FXN, PDHE1, ACTN1, MYL12A, ACTC1, and SORBS1 were nodal junctions; they interacted with each other and other proteins of the network. Nodes TF and HNRNPUL2 and NAA16 are outliers, referring to the fact that they don’t fall into any of the interacting groups, directly.

## Discussion

Clinical diagnosis of FRDA is still not common because of its complex phenotype, which overlaps with symptoms of other disorders such as Charcot-Marie-Tooth, ataxia with a Vitamin E deficiency, and others (Lynch and Farmer, 2002). Therefore, accurate diagnosis is crucial for better management of the disorder. Since no overlap has been seen between normal and affected individuals up to now, detection of the GAA repeats has become the basis of molecular diagnostic tests for FRDA^5^. Filla and co-workers described two methods to identify the expanded GAA repeats, firstly by PCR using intron1 primer and another by long-range PCR method (Filla et al., 1996). Lynch and co-workers further established the (GAA) expansion analysis method as the molecular diagnosis technique for FRDA (Brigatti et al., 2012). Long-range PCR has the advantage of amplifying the DNA lengths that cannot typically be amplified using routine PCR methods (up to 30 kb and beyond). This method employs a mixture of Taq polymerase and a thermostable DNA polymerase with proofreading activity, in a ratio optimized to generate a long amplicon with greater yield and higher fidelity.

Molecular analysis of genomic DNA confirmed that 25 out of all suspected patients carried GAA expansion in both the alleles of FXN gene in the pathogenic range and confirmed as homozygous for FRDA. The number of expanded (GAA) repeats in confirmed patients correlated with pathogenic range of (GAA) repeats established in FRDA homozygous patients (Campuzano et al., 1996). No alleles of intermediate size (34–100) were detected in this study. The normal-sized alleles were cantered around 1.5 kb, with a small range of 1.4–1.6 kb. The difference between normal and pathogenic allele was significantly large, with no overlap (1.4 and >4 kb bands), easily demarcating FRDA patients from healthy controls.

Genetic confirmation of FRDA was followed by assessment of its clinical severity by FARS. High FARS score indicates greater disability of FRDA patients. Ten homozygous patients confirmed in this study had high FARS scores (mean ± SD), 82.24 ± 12.02, which correlates with the severity of clinical symptoms observed in confirmed patients. Reliability of FARS score in assessing FRDA progression in patients is supported by many recent studies. Lynch and colleagues assessed 812 FRDA patients and reported that FARS outcome correlated with clinical measures of disease severity (Patel et al., 2016). Similar correlation results were previously reported by other studies as well (Fahey et al., 2007; Bürk et al., 2013), suggesting that FARS provides valid assessments of disease progression in FRDA.

All FRDA-confirmed patients showed symptoms of gait and limb ataxia; however, 80% of patients exhibited loss of lower-limb deep tendon reflexes. Areflexia was detected in 80% of patients, whereas dysarthria and nystagmus were accounted for in 90% and 88% of all FRDA patients, respectively. Mild to moderate degeneration of one or more regions of brain/spinal tract was observed in 32% of patients. Mild cardiomyopathy was observed in two patients; however, ECG in around 20% of patients revealed T-wave inversion. Diabetes mellitus was seen in only 1 patient in this study, given its later development in life.

### Relevance of Identified Proteins in FRDA

PBMCs are a heterogeneous cell population composed of lymphocytes (70–90%), monocytes (10–30%) and dendritic cells (1–2%). Understanding the changes in the proteome profiling of these blood cells can provide new insights into the pathogenesis of FRDA. Two-dimensional electrophoresis is one of the most commonly used techniques in proteomics since 1975 (O’farrell, 1975). This method has been used for more than three decades to study the different aspects of protein complement including biomarker studies (Bandow et al., 2008). However the limitations of conventional 2D-GE for applications like protein expression profiling in neuroscience research are the lack of reproducibility and quantitation. These shortcomings of this technique were addressed by introduction of two-dimensional difference gel electrophoresis (2D-DIGE) by Unlü et al. (1997). Since its introduction, 2D-DIGE is considered as one of the most useful techniques for quantitative analysis of the proteome. This method minimizes the gel-to-gel variation by allowing multiplexing of three different protein samples that can be run on a single 2-D gel, offering appropriate standardized quantitation in comparative proteomics (Freeman and Hemby, 2004). The Cy Dye (Cy2, Cy3, and Cy5) are the three fluorescent cyanine minimal dyes used to label proteins in the 2-D DIGE technique. They have similar mass and charge but differ in their excitation and emission wavelengths. Therefore, the same protein labeled with any of the Cy Dye DIGE Fluor minimal dyes will migrate to the same location on the 2-D gel. Further, use of internal control and dye swapping increases the sensitivity and reliability of this method.

The BVA module of Decyder directly provides the ratio of spot density of patients vs. healthy control after taking into account matching, quantification, and statistical analysis among all six gels. Four protein spots each were found to be up- and down-regulated in FRDA patients compared with controls as identified by LC-MS/MS and Mascot database search and confirmed by qPCR results.

### Down-Regulated Proteins

The identified down-regulated proteins with their function and their importance in relevance to FRDA is discussed in sequence.

#### ACTC1

Alpha cardiac actin is the major protein of the thin filament in cardiac sarcomeres that regulates the muscle contraction and pumping function of the heart. A significantly decreased level of ACTC1 (4.9-fold) in FRDA patients is seen in this study, which correlates with the observed mild cardiomyopathy and T wave inversion observed in around 20% patients. ACTC1 has previously been associated with various forms of cardiomyopathies (Frade et al., 2013; Maron et al., 2016).

#### PDHE1

PDHE1 is a part of the multienzyme complex enzyme pyruvate dehydrogenase (PDH) that converts pyruvate to acetyl-CoA and CO_2_, linking glycolysis to the Krebs cycle_._ Although the PDH complex plays an important role in all metabolically active tissues, it is critical for normal functioning of neurons under physiological conditions. This is because the brain usually obtains all of its energy from the aerobic oxidation of glucose (Imbard et al., 2011; Gray et al., 2014). PDH deficiency due to mutation or any cellular dysfunction leads to fatal lactic acidosis in the newborn and to a chronic neurodegenerative condition with gross structural abnormalities in the central nervous system (Imbard et al., 2011). Therefore, a 2.6-fold decrease in PDHE1 in FRDA patients clearly indicates its corresponding association with symptoms of ataxia and neurological abnormalities.

#### MYL12A

MYL12A plays a crucial role in regulating contractile activity of both smooth muscle and non-muscle cells via its phosphorylation. MYL12A is required for organization of stress fibers in interphase cells and the contractile ring in dividing cells (Iwasaki et al., 2001). Myosin regulatory light-chain protein defects have been associated with various neuromuscular disorders like sarcopenic muscle dysfunction (Gregorich et al., 2016). Therefore, low levels of this protein in FRDA patients may be one of the factors indicating the muscular defects seen in FRDA patients.

#### Alternate Protein HNRNPUL2

HNRNPUL2 stimulates double strand break (DSB) repair by homologous recombination, thereby promoting cell survival (Polo et al., 2012). These proteins are also believed to be associated with pre-mRNAs in the nucleus and appear to influence pre-mRNA processing and other aspects of mRNA metabolism and transport. This group of protein has been associated with mental retardation and Bain type X-linked syndromic, but its exact role in these pathologies is still under investigation (Bain et al., 2016). These proteins could not reveal a direct link with a particular pathology of FRDA, but we hypothesize that the decreased levels of these proteins could be associated with the increased mutations in this disease.

### Up-Regulated Proteins

The identified up-regulated proteins with their function and their importance in relevance to FRDA is discussed in sequence

#### CASP8

Caspases are a class of cysteine aspartate proteases that function as executioners of apoptosis. Apoptotic cell death associated with transcriptional elevated levels of CASP8 has been reported in various neurodegenerative diseases, including Alzheimer’s disease (AD) (Rehker et al., 2017), Parkinson’s disease (PD) (Hartmann et al., 2001), and stroke (Shabanzadeh et al., 2015). Therefore, the observed high levels of CASP8 in FRDA patients predict the increased neuroinflammation and neuronal death.

#### TF

It is well known that transferrins are iron binding proteins responsible for transporting iron from sites of absorption and heme degradation to those of storage and utilization. Up-regulated transferrin observed in this study explains the elevated cellular uptake of circulatory iron and its accumulation inside mitochondria, which is a hallmark characteristic of FRDA pathogenesis. This finding finds support from some previous studies reporting increased TF in FRDA patients (Wilson et al., 2000).

#### SORBS1

SORBS1 plays an important function in the signaling and stimulation of insulin-stimulated glucose transport. Involvement of SORBS1 has also been reported recently in diabetic nephropathy (Chang et al., 2018). Release of SORBS1 from damaged cardiac tissue into the bloodstream was observed in patients with acute myocardial infarction (Kakimoto et al., 2013). Therefore, the significant high levels of SORBS1 observed in patients, in the present study, perhaps indicate their corresponding association with diabetes and cardiac abnormalities in FRDA.

#### NAA16

NAA16 is the subunit of the N-terminal acetyltransferase A (NatA) complex that executes the N-terminal acetylation (NTA) modifications in proteins. Despite its role in NTA, very little is known about the precise biological function of NAA16. NTA has been linked to various diseases, including apoptosis and cancer (Kalvik and Arnesen, 2013). Perdiz et al. (2011) have reported that acetylated tubulin is crucial for neuronal polarization and neurite branching in the developing brain, hinting toward its possible role in neurodegeneration. Interestingly, some recent studies have shown that NTA might have a role in protein ubiquitination and degradation (Hwang et al., 2010). In the present study, we found high levels of NAA16 in PBMCs of FRDA patients, which are suggestive of their possible role in neurodegeneration as well as in frataxin degradation.

### PPI Analysis of the Differentially Expressed Proteins

Molecular processes in a cell are carried out by molecular machines composed of a large number of protein components organized by their PPIs. Understanding PPIs, therefore, is essential in gaining insights into the pathophysiology of any disease. Therefore, interaction of frataxin with eight differentially expressed proteins identified in this study was performed. As the precise cellular network of frataxin is not known, we first inquired about frataxin interacting proteins using BioGRID database and obtained four proteins LYR M4, PMPCB, RN126, and ACTN1. These are mainly mitochondrial proteins functioning in concert with frataxin in either Fe/S clusters assembly pathway or as chaperones. However, we felt that due to lack of availability of specific clustering algorithms and limited number of parameters offered in mapping the interactions, BioGRID could not be used for further PPI analysis. Therefore, STRING network analysis was applied to the eight differentially expressed proteins from proteomics study with frataxin and its interacting proteins (identified by BioGRID). Firstly, it can be seen from Figure 4 that all the query proteins are directly or indirectly making interactions with frataxin. Figure 4 illustrates the two network maps, the one with FXN, LYRM4, PMPCB, and RNF126 and the other with CASP8, SORBS1, MYL12A, ACTC1, and ACTN1 connected via PDHE1. Protein interacting with C kinase (PICK1) was included in the network by default (STRING) due to its interaction with one of the query proteins, MYL12A. A strong interaction observed between PDHE1 and FXN in the PPI network indicates the role of PDHE1 in connection with the reported brain anomalies like cerebral cortex atrophy and demyelination (Singhi et al., 2013), weak muscle tone and poor coordination (Debray et al., 2008), and cardiac complications (Gopal et al., 2018) in FRDA patients. The other interactions among ACTC1, ACTN1, MYL12A, and SORBS1 further suggest their correlation in several cellular pathways like insulin signaling and cross bridge cycling kinetics of skeletal and cardiac muscles, which are indeed compromised in FRDA (Figure 4).

All the above PPI clearly suggests that the differentially expressed proteins in PBMCs correlate well with a number of cellular pathways including iron metabolism, caspase, and glucose metabolism, actin filament-based movements in cardiac and skeletal muscles that have relevance in the pathogenesis of FRDA.

## Conclusion

The present study is the first report on the complete PBMC proteome in FRDA. Some of the differentially expressed proteins found in patients and their PPI analysis reveal their importance in understanding of the molecular events underlying the pathogenesis of FRDA. Proteins of special interest, PDHE1 and ATCTC1, found in this study open up new avenues in understanding the molecular mechanisms with respect to neurological and cardiac complications in FRDA. Further, we suggest that PDHE1 and ATCTC1 can find application in assessing the disease status of FRDA and also in therapeutic interventions.

## Data Availability Statement

The datasets generated for this study can be found in the PRIDE repository, accession number: 1-20190919-40510.

## Ethics Statement

The studies involving human participants were reviewed and approved by Ethical Clearance was obtained as per institutional ethical committee guidelines from AIIMS, New Delhi. Ethical Clearance No. IESC/T-45/21.01.2015. Written informed consent to participate in this study was provided by the participants’ legal guardian/next of kin.

## Author Contributions

MR conceived the idea, designed the experiments, and wrote the manuscript. MP, AS, and SG provided the FRDA patient samples. DP executed the experiments, acquired the raw data, and wrote the first draft of the manuscript. All authors were involved in interpreting the data and approved the final manuscript.

## Conflict of Interest

The authors declare that the research was conducted in the absence of any commercial or financial relationships that could be construed as a potential conflict of interest.

## Footnotes

1 www.thebiogrid.org

2 https://string-db.org/

3 https://www.ncbi.nlm.nih.gov

4 http://bioinfo.ut.ee/primer3-0.4.0/primer3

5 www.curefa.org
